# (2*R*,3*S*,4*R*)-3,4-Isopropyl­idenedi­oxy-2-(phenyl­sulfonyl­meth­yl)pyrrolidin-1-ol

**DOI:** 10.1107/S1600536812033028

**Published:** 2012-07-28

**Authors:** Mari Fe Flores, Pilar Garcia, Narciso M. Garrido, Francisca Sanz, David Diez

**Affiliations:** a Departamento de Quimica Organica, Universidad de Salamanca, Plaza de los Caidos, 37008 Salamanca, Spain; b Servicio General de Rayos X, Universidad de Salamanca, Plaza de los Caidos, 37008 Salamanca, Spain

## Abstract

The title compound, C_14_H_19_NO_5_S, was prepared by nucleophilic addition of the lithium derivative of methyl­phenyl­sulfone to (3*S*,4*R*)-3,4-isopropyl­idene­dioxy­pyrroline 1-oxide. There are four mol­ecules in the asymmetric unit. The crystal structure determination confirms the configuration of the chiral centres as 2*R*,3*S*,4*R*. In the crystal, pairs of O—H⋯N hydrogen bonds link the mol­ecules into dimers.

## Related literature

For asymmetric organocatalysis, see: Macmillan (2008 ▶); List (2007 ▶). For proline and its derivatives as organocatalysts, see: Pellissier (2007 ▶); Lattanzi (2009 ▶); Mielgo *et al.* (2008 ▶); Panday (2011 ▶). For the preparation, see: Flores *et al.* (2010 ▶). For C-branched pyrrolidines, see: Flores *et al.* (2011*a* ▶). For hydroxyl­amines in synthesis, see: Chevrier *et al.* (2011 ▶); Li *et al.* (2011 ▶). For (3*R*,4*S*)-3,4-isopropyl­idenedi­oxy-5-phenyl­sulf­on­yl­methyl-3,4-dihydro-2*H*-pyrrole 1-oxide, see: Flores *et al.* (2011*b* ▶).
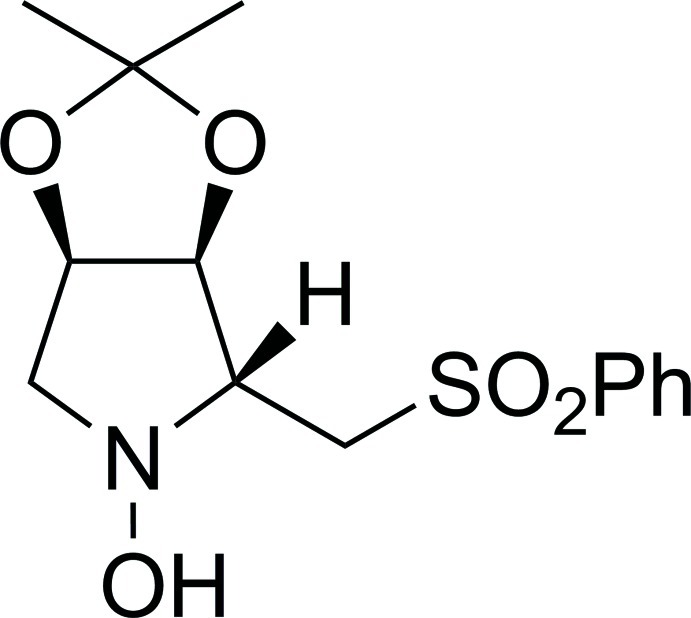


## Experimental

### 

#### Crystal data

C_14_H_19_NO_5_S
*M* *_r_* = 313.36
Monoclinic, 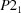

*a* = 9.1876 (2) Å
*b* = 19.5284 (5) Å
*c* = 17.9187 (5) Å
β = 102.658 (2)°
*V* = 3136.82 (14) Å^3^
*Z* = 8
Cu *K*α radiation
μ = 2.02 mm^−1^
*T* = 298 K
0.20 × 0.15 × 0.10 mm

#### Data collection

Bruker APEXII CCD area-detector diffractometer
Absorption correction: multi-scan (*SADABS*; Bruker, 2006 ▶) *T*_min_ = 0.730, *T*_max_ = 0.817
46376 measured reflections
10345 independent reflections
9616 reflections with *I* > 2σ(*I*)
*R*_int_ = 0.036

#### Refinement

*R*[*F* ^2^ > 2σ(*F* ^2^)] = 0.048
*wR*(*F* ^2^) = 0.135
*S* = 1.03
10345 reflections
769 parameters
1 restraint
H-atom parameters constrained
Δρ_max_ = 0.34 e Å^−3^
Δρ_min_ = −0.38 e Å^−3^
Absolute structure: Flack (1983 ▶), 4863 Friedel pairs
Flack parameter: 0.040 (14)

### 

Data collection: *APEX2* (Bruker, 2006 ▶); cell refinement: *SAINT* (Bruker, 2006 ▶); data reduction: *SAINT*; program(s) used to solve structure: *SHELXS97* (Sheldrick, 2008 ▶); program(s) used to refine structure: *SHELXL97* (Sheldrick, 2008 ▶); molecular graphics: *MERCURY* (Macrae *et al.*, 2006 ▶); software used to prepare material for publication: *SHELXTL* (Sheldrick, 2008 ▶).

## Supplementary Material

Crystal structure: contains datablock(s) global, I. DOI: 10.1107/S1600536812033028/bt5981sup1.cif

Structure factors: contains datablock(s) I. DOI: 10.1107/S1600536812033028/bt5981Isup2.hkl

Additional supplementary materials:  crystallographic information; 3D view; checkCIF report

## Figures and Tables

**Table 1:** Hydrogen-bond geometry (Å, °)

*D*—H⋯*A*	*D*—H	H⋯*A*	*D*⋯*A*	*D*—H⋯*A*
O5*A*—H5*AO*⋯N1*B* ^i^	0.82	2.04	2.752 (3)	144
O5*B*—H5*BO*⋯N1*A* ^ii^	0.82	2.11	2.807 (3)	142
O5*C*—H5*CO*⋯N1*D* ^i^	0.82	2.10	2.802 (4)	144
O5*D*—H5*DO*⋯N1*C* ^ii^	0.82	2.06	2.766 (3)	144

Symmetry codes: (i) 
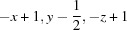
; (ii) 
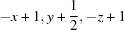
.

**Fig. 1.**
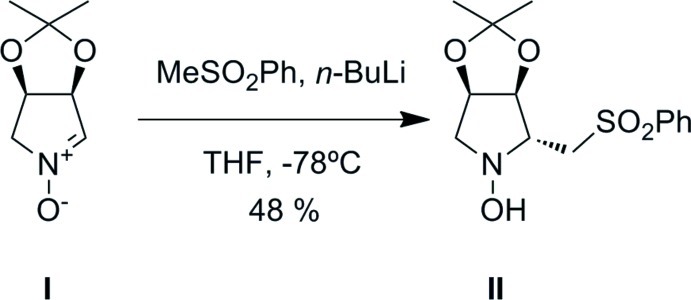
Reaction scheme.

**Fig. 2.**
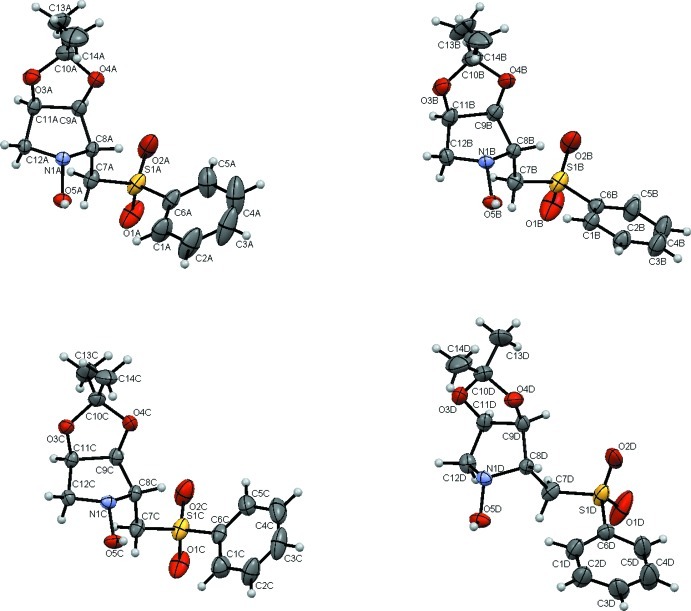
Views of molecules A, B, C and D with the atomic numbering schemes. Displacement ellipsoids are drawn at the 30% probability level. Hydrogen atoms are shown as spheres of arbitrary radius.

**Fig. 3.**
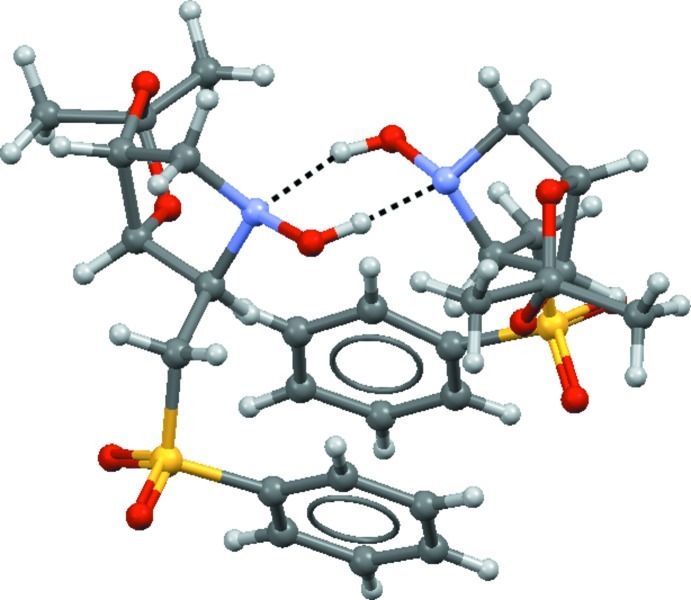
A view of the N—H···O hydrogen bonds in a dimer.

**Fig. 4.**
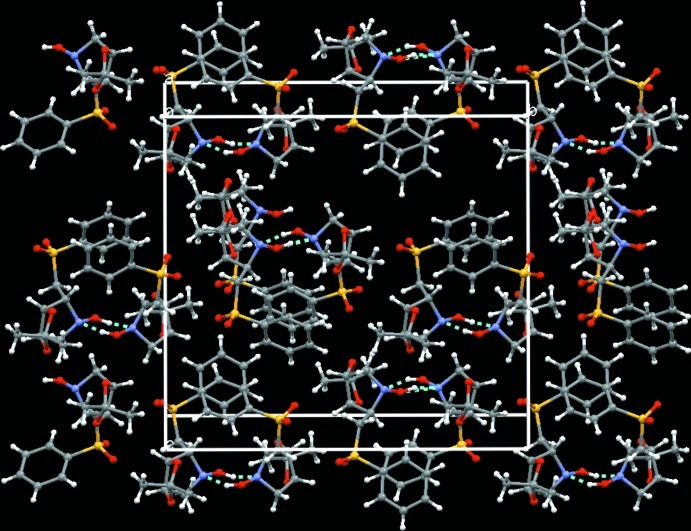
Crystal packing of C14H19NO5S viewed along a axis, showing intermolecular hydrogen bonding.

## supplementary crystallographic information

### Comment

Asymmetric organocatalysis has become a very attractive methodology in recent years, since environmentally friendly and metal-free transformations are desired (Macmillan, 2008; List, 2007). It is well known that proline and its derivatives have been widely employed as organocatalysts (Pellissier, 2007; Lattanzi, 2009; Mielgo *et al.* 2008; Panday, 2011). In our research group we have developed new organocatalysts using nitrones as starting material (Flores *et al.*, 2010). This catalyst was obtained from the reduction of the chiral hydroxylamine (II) (Fig. 1). Moreover, this hydroxylamine has been employed as starting material to tackle the synthesis of a C-branched substituted pyrrolidine (Flores *et al.*, 2011*a*). Hydroxylamines are important tools for the synthesis of biologically active compounds (Chevrier *et al.*, 2011; Li *et al.*, 2011).

The title compound, C_14_H_19_NO_5_S, consists of a *N*-hydroxypyrrolidine ring with a phenylsulfonylmethyl group and an isopropylidenedioxy group as susbtituents. This compound crystallizes in space group *P*2_1_ with four independent molecules (A, B, C and D) in the asymmetric unit which differ slightly in conformation but retain the same (*3S,4R*) configuration in the acetonide group (Fig. 2). All the bond lengths and angles are within the normal ranges. The C—S—C angles in molecules A, B, C and D are 108.5 (2)°, 104.9 (2)°, 106.4 (2)° and 106.1 (2)°, respectively and the O—S—O angles are 115.6 (3)°, 119.8 (2)°, 116.9 (3)° and 118.3 (2)°, respectively. The large O—S—O angle and this deviation from the optimal 109.5° angle can be explained by the repulsion of the lone pairs of the oxygen placing the oxygen atoms as far away from each other as possible and thus minimizing the C—S—C angle. Torsion-angle differences in molecules A, B, C and D are evident from C6—S1—C7—C8, with values of 78.6 (3)°, 60.6 (3)°, 81.6 (3)° and 60.2 (3)°, respectively. The hydroxyl group at N1 atom is displaced from the planar conformation with the pyrrolidine ring. The O5—N1—C8—C9 torsion angles are very similar in the four molecules, having values of 165.3 (2)°, 164.6 (2)°, 166.0 (2)° and 163.5 (2)°, respectively.

In the crystal, O—H···N hydrogen bonds between the hydroxyl group and the nitrogen atom of the *N*-hydroxypyrrolidine link adjacent molecules into dimers (Table 1 and Fig. 3). The dimers form six-membered rings and present an anti-parallel orientation along the [010] direction, as is shown in the crystal packing (Fig. 4).

The structure of (3R,4S)-3,4-Isopropylidenedioxy-5-phenylsulfonylmethyl-3,4-dihydro-2H-pyrrole 1-oxide has been determined by Flores *et al.* (2011*b*).

### Experimental

The title hydroxylamine, (II), was obtained stereoselectively by nucleophilic addition of lithio(phenylsulfonyl)methane to (*3S,4R*)-3,4-isopropylidenedioxypyrroline-1-oxide (I) according to the methodology described by us (Flores *et al.* 2010). The stereochemistry of (II) was established studying its NMR spectra and observation of the nOes that this molecule displays. The X-ray analysis corroborated its configuration. Well shaped colourless single crystals were obtained by crystallization from hexane/EtOAc.

### Refinement

The hydrogen atoms were positioned geometrically, with C—H distances constrained to 0.93 Å (aromatic), 0.96 Å (methyl), 0.97 Å (methylene) and refined in riding mode with *U*_iso_(H) = xUeq(C), where *x* = 1.5 for methyl H atoms and *x* = 1.2 for all other H atoms. The hydroxyl hydrogen atoms were positined with an O—H distance of 0.82 Å, starting from the difference Fourier map coordinates and with *U*_iso_(H) = 1.5 *U*_eq_(O).

### Crystal data

**Table:**

C_14_H_19_NO_5_S	*F*(000) = 1328
*M**_r_* = 313.36	*D*_x_ = 1.327 Mg m^−^^3^
Monoclinic, *P*2_1_	Cu *K*α radiation, λ = 1.54178 Å
Hall symbol: P 2yb	Cell parameters from 9406 reflections
*a* = 9.1876 (2) Å	θ = 4.9–63.6°
*b* = 19.5284 (5) Å	µ = 2.02 mm^−^^1^
*c* = 17.9187 (5) Å	*T* = 298 K
β = 102.658 (2)°	Monoclinic, colorless
*V* = 3136.82 (14) Å^3^	0.20 × 0.15 × 0.10 mm
*Z* = 8	

### Data collection

**Table:**

Bruker APEXII CCD area-detector diffractometer	10345 independent reflections
Radiation source: fine-focus sealed tube	9616 reflections with *I* > 2σ(*I*)
Graphite monochromator	*R*_int_ = 0.036
phi and ω scans	θ_max_ = 67.3°, θ_min_ = 4.5°
Absorption correction: multi-scan (*SADABS*; Bruker, 2006)	*h* = −10→9
*T*_min_ = 0.730, *T*_max_ = 0.817	*k* = −22→22
46376 measured reflections	*l* = −19→21

### Refinement

**Table:**

Refinement on *F*^2^	Secondary atom site location: difference Fourier map
Least-squares matrix: full	Hydrogen site location: inferred from neighbouring sites
*R*[*F*^2^ > 2σ(*F*^2^)] = 0.048	H-atom parameters constrained
*wR*(*F*^2^) = 0.135	*w* = 1/[σ^2^(*F*_o_^2^) + (0.0765*P*)^2^ + 1.156*P*] where *P* = (*F*_o_^2^ + 2*F*_c_^2^)/3
*S* = 1.03	(Δ/σ)_max_ < 0.001
10345 reflections	Δρ_max_ = 0.34 e Å^−^^3^
769 parameters	Δρ_min_ = −0.38 e Å^−^^3^
1 restraint	Absolute structure: Flack (1983), 4863 Friedel pairs
Primary atom site location: structure-invariant direct methods	Flack parameter: 0.040 (14)

### Special details

**Table:**

Geometry. All e.s.d.'s (except the e.s.d. in the dihedral angle between two l.s. planes) are estimated using the full covariance matrix. The cell e.s.d.'s are taken into account individually in the estimation of e.s.d.'s in distances, angles and torsion angles; correlations between e.s.d.'s in cell parameters are only used when they are defined by crystal symmetry. An approximate (isotropic) treatment of cell e.s.d.'s is used for estimating e.s.d.'s involving l.s. planes.
Refinement. Refinement of *F*^2^ against ALL reflections. The weighted *R*-factor *wR* and goodness of fit *S* are based on *F*^2^, conventional *R*-factors *R* are based on *F*, with *F* set to zero for negative *F*^2^. The threshold expression of *F*^2^ > σ(*F*^2^) is used only for calculating *R*-factors(gt) *etc*. and is not relevant to the choice of reflections for refinement. *R*-factors based on *F*^2^ are statistically about twice as large as those based on *F*, and *R*- factors based on ALL data will be even larger.

### Fractional atomic coordinates and isotropic or equivalent isotropic displacement parameters (Å^2^)

**Table:**

	*x*	*y*	*z*	*U*_iso_*/*U*_eq_	
S1A	−0.19647 (12)	−0.61777 (4)	0.05085 (6)	0.0652 (2)	
O1A	−0.3193 (6)	−0.6595 (2)	0.0564 (3)	0.1277 (16)	
O2A	−0.0537 (5)	−0.64490 (18)	0.08459 (18)	0.1012 (12)	
O3A	0.1249 (3)	−0.62313 (16)	−0.19626 (14)	0.0765 (8)	
O4A	0.1892 (3)	−0.60587 (18)	−0.06966 (15)	0.0759 (8)	
O5A	−0.2162 (2)	−0.49040 (11)	−0.14264 (14)	0.0548 (5)	
H5AO	−0.1900	−0.4508	−0.1472	0.082*	
N1A	−0.0905 (3)	−0.53544 (12)	−0.13787 (14)	0.0436 (5)	
C1A	−0.2376 (4)	−0.47657 (19)	0.0578 (2)	0.0601 (9)	
H1A	−0.2360	−0.4738	0.0062	0.072*	
C2A	−0.2580 (5)	−0.4184 (2)	0.0980 (3)	0.0755 (11)	
H2A	−0.2682	−0.3760	0.0737	0.091*	
C3A	−0.2633 (7)	−0.4228 (3)	0.1733 (3)	0.1013 (17)	
H3A	−0.2816	−0.3837	0.1995	0.122*	
C4A	−0.2419 (8)	−0.4842 (3)	0.2107 (3)	0.114 (2)	
H4A	−0.2421	−0.4864	0.2625	0.137*	
C5A	−0.2199 (7)	−0.5428 (3)	0.1721 (2)	0.0944 (15)	
H5A	−0.2055	−0.5846	0.1975	0.113*	
C6A	−0.2196 (4)	−0.53896 (18)	0.09506 (19)	0.0548 (8)	
C7A	−0.2040 (4)	−0.60265 (18)	−0.0472 (2)	0.0563 (8)	
H7A1	−0.2274	−0.6454	−0.0747	0.068*	
H7A2	−0.2849	−0.5710	−0.0663	0.068*	
C8A	−0.0619 (3)	−0.57383 (15)	−0.06490 (17)	0.0438 (6)	
H8A	−0.0101	−0.5448	−0.0228	0.053*	
C9A	0.0419 (4)	−0.62954 (17)	−0.08194 (19)	0.0556 (8)	
H9A	0.0336	−0.6720	−0.0540	0.067*	
C10A	0.2494 (5)	−0.6259 (3)	−0.1332 (2)	0.0866 (15)	
C11A	−0.0040 (4)	−0.63964 (18)	−0.1709 (2)	0.0604 (9)	
H11A	−0.0382	−0.6863	−0.1850	0.072*	
C12A	−0.1243 (4)	−0.58701 (17)	−0.19824 (19)	0.0567 (8)	
H12A	−0.1174	−0.5681	−0.2474	0.068*	
H12B	−0.2229	−0.6063	−0.2021	0.068*	
C13A	0.3073 (9)	−0.6989 (4)	−0.1226 (4)	0.159 (4)	
H13A	0.2379	−0.7267	−0.1030	0.238*	
H13B	0.4023	−0.6994	−0.0873	0.238*	
H13C	0.3181	−0.7169	−0.1710	0.238*	
C14A	0.3633 (6)	−0.5742 (5)	−0.1443 (4)	0.138 (3)	
H14A	0.4043	−0.5873	−0.1871	0.207*	
H14B	0.4417	−0.5721	−0.0990	0.207*	
H14C	0.3170	−0.5300	−0.1538	0.207*	
S1B	0.74157 (11)	0.17691 (4)	0.93555 (5)	0.0663 (3)	
O1B	0.5883 (4)	0.19608 (18)	0.9256 (2)	0.0996 (11)	
O2B	0.8381 (5)	0.21718 (17)	0.90313 (18)	0.1016 (11)	
O3B	1.2830 (2)	0.19731 (12)	1.17829 (11)	0.0503 (5)	
O4B	1.2291 (3)	0.17175 (14)	1.05340 (13)	0.0614 (6)	
O5B	0.8899 (2)	0.07129 (12)	1.14583 (16)	0.0598 (6)	
H5BO	0.9183	0.0315	1.1527	0.090*	
N1B	1.0105 (3)	0.11334 (12)	1.13398 (14)	0.0431 (5)	
C1B	0.6650 (6)	0.0428 (2)	0.9269 (3)	0.0841 (12)	
H1B	0.6067	0.0545	0.9614	0.101*	
C2B	0.6667 (8)	−0.0242 (3)	0.9021 (3)	0.1065 (18)	
H2B	0.6148	−0.0582	0.9217	0.128*	
C3B	0.7454 (10)	−0.0391 (4)	0.8490 (4)	0.124 (3)	
H3B	0.7418	−0.0833	0.8294	0.149*	
C4B	0.8280 (7)	0.0079 (4)	0.8240 (4)	0.114 (2)	
H4B	0.8832	−0.0042	0.7883	0.137*	
C5B	0.8330 (6)	0.0757 (3)	0.8509 (3)	0.0877 (13)	
H5B	0.8930	0.1083	0.8345	0.105*	
C6B	0.7480 (4)	0.09241 (19)	0.9013 (2)	0.0576 (8)	
C7B	0.8145 (4)	0.17107 (19)	1.03508 (19)	0.0558 (8)	
H7B1	0.8104	0.2160	1.0576	0.067*	
H7B2	0.7516	0.1407	1.0570	0.067*	
C8B	0.9762 (3)	0.14469 (15)	1.05684 (16)	0.0430 (6)	
H8B	0.9930	0.1112	1.0189	0.052*	
C9B	1.0931 (4)	0.19970 (16)	1.06625 (16)	0.0472 (7)	
H9B	1.0599	0.2398	1.0341	0.057*	
C10B	1.3488 (4)	0.1946 (2)	1.1131 (2)	0.0613 (9)	
C11B	1.1306 (3)	0.21753 (15)	1.15338 (16)	0.0455 (7)	
H11B	1.1152	0.2661	1.1630	0.055*	
C12B	1.0312 (3)	0.17174 (16)	1.18733 (16)	0.0450 (6)	
H12C	1.0789	0.1577	1.2388	0.054*	
H12D	0.9370	0.1938	1.1882	0.054*	
C13B	1.3996 (6)	0.2649 (3)	1.0944 (3)	0.0967 (17)	
H13D	1.4503	0.2614	1.0531	0.145*	
H13E	1.4662	0.2835	1.1386	0.145*	
H13F	1.3145	0.2943	1.0796	0.145*	
C14B	1.4696 (5)	0.1411 (3)	1.1277 (3)	0.0930 (15)	
H14D	1.4273	0.0975	1.1358	0.140*	
H14E	1.5439	0.1534	1.1723	0.140*	
H14F	1.5149	0.1382	1.0843	0.140*	
S1C	0.25570 (12)	−0.15006 (5)	0.44754 (7)	0.0776 (3)	
O1C	0.1349 (5)	−0.1036 (2)	0.4415 (3)	0.145 (2)	
O2C	0.3711 (5)	−0.13160 (18)	0.40943 (19)	0.1102 (13)	
O3C	0.8029 (3)	−0.14324 (16)	0.68788 (15)	0.0760 (8)	
O4C	0.7499 (3)	−0.16513 (15)	0.56239 (16)	0.0707 (7)	
O5C	0.4103 (3)	−0.27178 (12)	0.64572 (17)	0.0641 (6)	
H5CO	0.4341	−0.3119	0.6418	0.096*	
N1C	0.5301 (3)	−0.22795 (12)	0.63647 (15)	0.0463 (6)	
C1C	0.2105 (5)	−0.2920 (2)	0.4527 (3)	0.0880 (14)	
H1C	0.2700	−0.2928	0.5019	0.106*	
C2C	0.1503 (6)	−0.3513 (2)	0.4191 (3)	0.1004 (17)	
H2C	0.1715	−0.3928	0.4447	0.120*	
C3C	0.0603 (8)	−0.3497 (3)	0.3490 (3)	0.116 (2)	
H3C	0.0260	−0.3905	0.3247	0.139*	
C4C	0.0181 (10)	−0.2883 (4)	0.3127 (4)	0.155 (3)	
H4C	−0.0499	−0.2874	0.2659	0.186*	
C5C	0.0775 (8)	−0.2287 (3)	0.3464 (3)	0.121 (2)	
H5C	0.0458	−0.1869	0.3236	0.145*	
C6C	0.1829 (4)	−0.2301 (2)	0.4133 (2)	0.0645 (9)	
C7C	0.3370 (4)	−0.16135 (19)	0.5455 (2)	0.0603 (9)	
H7C1	0.3409	−0.1173	0.5708	0.072*	
H7C2	0.2728	−0.1909	0.5676	0.072*	
C8C	0.4938 (3)	−0.19196 (15)	0.56218 (17)	0.0453 (7)	
H8C	0.5059	−0.2227	0.5208	0.054*	
C9C	0.6151 (4)	−0.13780 (16)	0.57601 (18)	0.0530 (8)	
H9C	0.5845	−0.0960	0.5465	0.064*	
C10C	0.8674 (5)	−0.1434 (3)	0.6235 (3)	0.0810 (13)	
C11C	0.6494 (4)	−0.12466 (17)	0.6639 (2)	0.0606 (9)	
H11C	0.6304	−0.0772	0.6766	0.073*	
C12C	0.5546 (4)	−0.17463 (18)	0.6950 (2)	0.0582 (8)	
H12E	0.6064	−0.1925	0.7442	0.070*	
H12F	0.4613	−0.1540	0.7002	0.070*	
C13C	0.9889 (6)	−0.1981 (4)	0.6367 (5)	0.128 (3)	
H13G	1.0709	−0.1833	0.6764	0.192*	
H13H	1.0230	−0.2052	0.5903	0.192*	
H13I	0.9494	−0.2402	0.6517	0.192*	
C14C	0.9201 (8)	−0.0723 (4)	0.6074 (4)	0.127 (2)	
H14G	0.8382	−0.0408	0.6013	0.190*	
H14H	0.9572	−0.0733	0.5613	0.190*	
H14I	0.9982	−0.0579	0.6493	0.190*	
S1D	0.32357 (13)	0.05362 (4)	0.57451 (5)	0.0674 (3)	
O1D	0.1838 (5)	0.03538 (18)	0.5906 (2)	0.1052 (12)	
O2D	0.4457 (5)	0.00924 (17)	0.59922 (17)	0.1037 (12)	
O3D	0.5969 (3)	0.03630 (14)	0.30822 (13)	0.0653 (7)	
O4D	0.6770 (3)	0.05050 (17)	0.43411 (15)	0.0727 (7)	
O5D	0.2675 (2)	0.16902 (11)	0.37198 (13)	0.0501 (5)	
H5DO	0.2918	0.2084	0.3644	0.075*	
N1D	0.3916 (3)	0.12378 (11)	0.37304 (13)	0.0400 (5)	
C1D	0.2912 (8)	0.1919 (3)	0.5823 (3)	0.1070 (18)	
H1D	0.2136	0.1873	0.5396	0.128*	
C2D	0.3254 (11)	0.2554 (3)	0.6154 (4)	0.143 (3)	
H2D	0.2721	0.2942	0.5951	0.172*	
C3D	0.4400 (14)	0.2599 (4)	0.6787 (4)	0.170 (4)	
H3D	0.4621	0.3022	0.7025	0.204*	
C4D	0.5208 (14)	0.2051 (5)	0.7073 (4)	0.182 (4)	
H4D	0.5994	0.2101	0.7496	0.218*	
C5D	0.4885 (9)	0.1410 (3)	0.6747 (3)	0.127 (2)	
H5D	0.5447	0.1029	0.6945	0.152*	
C6D	0.3732 (5)	0.13519 (18)	0.6132 (2)	0.0682 (10)	
C7D	0.2956 (4)	0.06272 (18)	0.47465 (18)	0.0545 (8)	
H7D1	0.2646	0.0189	0.4510	0.065*	
H7D2	0.2147	0.0949	0.4575	0.065*	
C8D	0.4322 (3)	0.08745 (15)	0.44659 (16)	0.0412 (6)	
H8D	0.4931	0.1173	0.4852	0.049*	
C9D	0.5275 (4)	0.02939 (16)	0.42674 (17)	0.0483 (7)	
H9D	0.5194	−0.0123	0.4559	0.058*	
C10D	0.7248 (4)	0.0301 (2)	0.3660 (2)	0.0712 (10)	
C11D	0.4722 (4)	0.01849 (16)	0.33885 (17)	0.0511 (7)	
H11D	0.4387	−0.0286	0.3261	0.061*	
C12D	0.3484 (4)	0.06992 (15)	0.31499 (17)	0.0467 (7)	
H12G	0.3452	0.0873	0.2639	0.056*	
H12H	0.2522	0.0504	0.3168	0.056*	
C13D	0.7763 (7)	−0.0446 (3)	0.3751 (3)	0.1072 (19)	
H13J	0.7017	−0.0717	0.3913	0.161*	
H13K	0.8682	−0.0474	0.4128	0.161*	
H13L	0.7912	−0.0614	0.3271	0.161*	
C14D	0.8396 (6)	0.0794 (4)	0.3508 (5)	0.127 (2)	
H14J	0.8802	0.0628	0.3092	0.191*	
H14K	0.9180	0.0838	0.3958	0.191*	
H14L	0.7942	0.1233	0.3376	0.191*	

### Atomic displacement parameters (Å^2^)

**Table:**

	*U*^11^	*U*^22^	*U*^33^	*U*^12^	*U*^13^	*U*^23^
S1A	0.0921 (7)	0.0466 (4)	0.0666 (5)	−0.0044 (4)	0.0387 (5)	0.0031 (4)
O1A	0.182 (4)	0.095 (3)	0.138 (3)	−0.074 (3)	0.104 (3)	−0.029 (2)
O2A	0.153 (3)	0.084 (2)	0.0693 (18)	0.063 (2)	0.0301 (19)	0.0204 (16)
O3A	0.0891 (19)	0.096 (2)	0.0490 (13)	0.0356 (16)	0.0249 (13)	0.0121 (13)
O4A	0.0602 (15)	0.113 (2)	0.0548 (14)	0.0340 (15)	0.0123 (11)	0.0012 (14)
O5A	0.0446 (11)	0.0444 (11)	0.0738 (14)	0.0118 (9)	0.0093 (10)	0.0071 (11)
N1A	0.0443 (13)	0.0385 (12)	0.0464 (13)	0.0101 (10)	0.0062 (10)	0.0048 (10)
C1A	0.077 (2)	0.053 (2)	0.0553 (19)	0.0063 (17)	0.0243 (17)	0.0004 (16)
C2A	0.101 (3)	0.057 (2)	0.072 (3)	0.012 (2)	0.027 (2)	−0.0054 (19)
C3A	0.155 (5)	0.080 (3)	0.073 (3)	0.019 (3)	0.035 (3)	−0.021 (3)
C4A	0.189 (6)	0.107 (4)	0.055 (2)	0.023 (4)	0.045 (3)	−0.011 (3)
C5A	0.153 (5)	0.081 (3)	0.053 (2)	0.020 (3)	0.032 (3)	0.009 (2)
C6A	0.061 (2)	0.0546 (19)	0.0514 (18)	0.0075 (15)	0.0185 (15)	0.0015 (15)
C7A	0.0594 (19)	0.0469 (18)	0.065 (2)	−0.0071 (14)	0.0184 (16)	−0.0080 (15)
C8A	0.0474 (16)	0.0421 (15)	0.0421 (15)	0.0039 (12)	0.0104 (12)	0.0034 (12)
C9A	0.071 (2)	0.0517 (18)	0.0472 (17)	0.0183 (15)	0.0192 (15)	0.0140 (14)
C10A	0.080 (3)	0.125 (4)	0.062 (2)	0.056 (3)	0.031 (2)	0.024 (2)
C11A	0.080 (2)	0.0460 (18)	0.0578 (19)	0.0129 (16)	0.0204 (17)	−0.0057 (15)
C12A	0.068 (2)	0.0527 (19)	0.0450 (16)	0.0023 (16)	0.0022 (15)	−0.0037 (14)
C13A	0.199 (7)	0.198 (7)	0.091 (4)	0.157 (7)	0.059 (4)	0.043 (4)
C14A	0.068 (3)	0.225 (9)	0.132 (5)	0.017 (4)	0.045 (3)	0.031 (5)
S1B	0.0772 (6)	0.0489 (5)	0.0603 (5)	0.0084 (4)	−0.0121 (4)	−0.0012 (4)
O1B	0.084 (2)	0.093 (2)	0.100 (2)	0.0439 (17)	−0.0280 (16)	−0.0245 (18)
O2B	0.145 (3)	0.080 (2)	0.0660 (17)	−0.030 (2)	−0.0066 (18)	0.0094 (16)
O3B	0.0491 (12)	0.0598 (13)	0.0417 (10)	−0.0069 (9)	0.0093 (9)	0.0027 (9)
O4B	0.0612 (14)	0.0806 (16)	0.0475 (11)	−0.0209 (12)	0.0227 (10)	−0.0118 (12)
O5B	0.0470 (12)	0.0517 (13)	0.0858 (17)	−0.0083 (9)	0.0259 (12)	0.0049 (12)
N1B	0.0422 (13)	0.0404 (13)	0.0487 (13)	−0.0034 (10)	0.0144 (10)	0.0029 (10)
C1B	0.096 (3)	0.070 (3)	0.090 (3)	−0.005 (2)	0.028 (2)	−0.011 (2)
C2B	0.141 (5)	0.074 (3)	0.093 (4)	−0.024 (3)	0.001 (3)	−0.017 (3)
C3B	0.173 (7)	0.082 (4)	0.094 (4)	0.030 (4)	−0.025 (4)	−0.037 (3)
C4B	0.111 (4)	0.133 (5)	0.096 (4)	0.029 (4)	0.018 (3)	−0.056 (4)
C5B	0.085 (3)	0.104 (4)	0.075 (3)	0.001 (3)	0.020 (2)	−0.022 (2)
C6B	0.0563 (19)	0.0568 (19)	0.0513 (18)	0.0067 (15)	−0.0067 (15)	−0.0052 (15)
C7B	0.0544 (18)	0.0526 (18)	0.0549 (18)	0.0076 (15)	−0.0001 (14)	−0.0086 (15)
C8B	0.0462 (15)	0.0395 (14)	0.0414 (15)	−0.0006 (12)	0.0056 (12)	−0.0070 (12)
C9B	0.0568 (18)	0.0470 (16)	0.0369 (14)	−0.0018 (13)	0.0081 (12)	0.0073 (12)
C10B	0.0557 (19)	0.079 (2)	0.0515 (17)	−0.0242 (17)	0.0178 (15)	−0.0005 (16)
C11B	0.0563 (17)	0.0384 (15)	0.0393 (14)	−0.0010 (13)	0.0053 (12)	−0.0062 (12)
C12B	0.0490 (16)	0.0460 (15)	0.0400 (14)	0.0043 (13)	0.0101 (12)	−0.0018 (13)
C13B	0.117 (4)	0.105 (4)	0.073 (3)	−0.068 (3)	0.031 (2)	0.001 (2)
C14B	0.056 (2)	0.126 (4)	0.107 (4)	−0.004 (2)	0.038 (2)	−0.003 (3)
S1C	0.0818 (7)	0.0426 (5)	0.0882 (7)	0.0055 (4)	−0.0254 (5)	0.0047 (5)
O1C	0.130 (3)	0.094 (3)	0.165 (4)	0.063 (2)	−0.068 (3)	−0.028 (3)
O2C	0.147 (3)	0.090 (2)	0.0768 (19)	−0.055 (2)	−0.012 (2)	0.0305 (17)
O3C	0.0683 (16)	0.088 (2)	0.0619 (15)	−0.0182 (14)	−0.0078 (12)	0.0126 (14)
O4C	0.0583 (15)	0.084 (2)	0.0704 (16)	−0.0222 (13)	0.0149 (12)	−0.0018 (13)
O5C	0.0557 (13)	0.0464 (12)	0.0955 (18)	−0.0001 (10)	0.0283 (13)	0.0059 (13)
N1C	0.0506 (14)	0.0362 (12)	0.0523 (14)	−0.0023 (10)	0.0119 (11)	0.0035 (10)
C1C	0.094 (3)	0.063 (3)	0.088 (3)	−0.005 (2)	−0.022 (2)	0.003 (2)
C2C	0.109 (4)	0.059 (2)	0.114 (4)	−0.015 (2)	−0.019 (3)	0.012 (3)
C3C	0.167 (6)	0.085 (3)	0.077 (3)	−0.057 (4)	−0.013 (3)	0.006 (3)
C4C	0.226 (8)	0.116 (5)	0.084 (4)	−0.065 (5)	−0.053 (4)	0.024 (3)
C5C	0.171 (6)	0.085 (3)	0.079 (3)	−0.042 (4)	−0.035 (3)	0.030 (3)
C6C	0.062 (2)	0.057 (2)	0.068 (2)	−0.0115 (16)	0.0003 (17)	0.0035 (17)
C7C	0.0513 (18)	0.051 (2)	0.074 (2)	0.0101 (15)	0.0025 (16)	−0.0061 (16)
C8C	0.0467 (17)	0.0404 (15)	0.0460 (16)	−0.0004 (12)	0.0041 (12)	−0.0025 (12)
C9C	0.064 (2)	0.0427 (17)	0.0467 (16)	−0.0060 (14)	0.0005 (14)	0.0052 (13)
C10C	0.061 (2)	0.088 (3)	0.086 (3)	−0.027 (2)	−0.002 (2)	0.016 (2)
C11C	0.080 (2)	0.0399 (16)	0.0526 (18)	−0.0041 (15)	−0.0064 (16)	−0.0023 (14)
C12C	0.076 (2)	0.0495 (18)	0.0500 (18)	0.0105 (16)	0.0152 (16)	−0.0039 (14)
C13C	0.048 (3)	0.142 (6)	0.185 (7)	−0.008 (3)	0.006 (3)	0.022 (5)
C14C	0.133 (5)	0.124 (5)	0.113 (4)	−0.080 (4)	0.004 (4)	0.018 (4)
S1D	0.1189 (8)	0.0413 (4)	0.0509 (4)	0.0020 (5)	0.0376 (5)	0.0055 (4)
O1D	0.156 (3)	0.092 (2)	0.093 (2)	−0.046 (2)	0.081 (2)	−0.0096 (18)
O2D	0.180 (4)	0.0728 (19)	0.0583 (16)	0.045 (2)	0.0269 (19)	0.0101 (14)
O3D	0.0701 (15)	0.0782 (17)	0.0527 (12)	0.0171 (13)	0.0243 (12)	0.0067 (12)
O4D	0.0553 (14)	0.097 (2)	0.0608 (14)	0.0177 (13)	0.0025 (11)	−0.0193 (14)
O5D	0.0487 (11)	0.0422 (11)	0.0606 (12)	0.0061 (9)	0.0143 (9)	0.0057 (10)
N1D	0.0470 (13)	0.0344 (12)	0.0377 (12)	0.0020 (9)	0.0076 (10)	0.0020 (9)
C1D	0.164 (5)	0.057 (3)	0.096 (4)	0.019 (3)	0.019 (3)	−0.012 (2)
C2D	0.287 (10)	0.049 (3)	0.103 (5)	0.008 (4)	0.062 (6)	−0.010 (3)
C3D	0.367 (14)	0.075 (4)	0.079 (4)	−0.048 (6)	0.067 (6)	−0.017 (3)
C4D	0.292 (12)	0.132 (7)	0.091 (5)	−0.068 (7)	−0.024 (6)	−0.011 (5)
C5D	0.195 (7)	0.092 (4)	0.069 (3)	−0.022 (4)	−0.022 (4)	0.000 (3)
C6D	0.113 (3)	0.0465 (19)	0.054 (2)	0.0021 (19)	0.037 (2)	−0.0034 (15)
C7D	0.069 (2)	0.0500 (18)	0.0480 (16)	−0.0104 (15)	0.0215 (15)	−0.0006 (14)
C8D	0.0497 (16)	0.0391 (14)	0.0346 (13)	−0.0017 (12)	0.0091 (11)	−0.0008 (11)
C9D	0.0622 (19)	0.0439 (16)	0.0379 (14)	0.0088 (13)	0.0088 (13)	0.0076 (12)
C10D	0.065 (2)	0.082 (3)	0.066 (2)	0.020 (2)	0.0156 (18)	−0.004 (2)
C11D	0.066 (2)	0.0420 (16)	0.0450 (16)	0.0021 (14)	0.0109 (14)	−0.0063 (13)
C12D	0.0551 (17)	0.0465 (16)	0.0364 (14)	−0.0018 (13)	0.0057 (12)	−0.0039 (12)
C13D	0.127 (4)	0.109 (4)	0.084 (3)	0.073 (3)	0.021 (3)	0.002 (3)
C14D	0.073 (3)	0.163 (6)	0.156 (6)	−0.006 (4)	0.049 (4)	−0.007 (5)

### Geometric parameters (Å, º)

**Table:**

S1A—O1A	1.414 (3)	S1C—O1C	1.419 (4)
S1A—O2A	1.421 (4)	S1C—O2C	1.427 (4)
S1A—C6A	1.765 (4)	S1C—C6C	1.757 (4)
S1A—C7A	1.767 (4)	S1C—C7C	1.764 (4)
O3A—C11A	1.395 (5)	O3C—C10C	1.407 (6)
O3A—C10A	1.422 (5)	O3C—C11C	1.428 (5)
O4A—C9A	1.401 (5)	O4C—C9C	1.418 (4)
O4A—C10A	1.425 (5)	O4C—C10C	1.424 (5)
O5A—N1A	1.439 (3)	O5C—N1C	1.432 (3)
O5A—H5AO	0.8200	O5C—H5CO	0.8200
N1A—C12A	1.461 (4)	N1C—C12C	1.460 (4)
N1A—C8A	1.480 (4)	N1C—C8C	1.477 (4)
C1A—C2A	1.380 (5)	C1C—C2C	1.366 (7)
C1A—C6A	1.382 (5)	C1C—C6C	1.394 (6)
C1A—H1A	0.9300	C1C—H1C	0.9300
C2A—C3A	1.363 (7)	C2C—C3C	1.345 (7)
C2A—H2A	0.9300	C2C—H2C	0.9300
C3A—C4A	1.366 (8)	C3C—C4C	1.379 (9)
C3A—H3A	0.9300	C3C—H3C	0.9300
C4A—C5A	1.374 (7)	C4C—C5C	1.367 (8)
C4A—H4A	0.9300	C4C—H4C	0.9300
C5A—C6A	1.383 (5)	C5C—C6C	1.367 (6)
C5A—H5A	0.9300	C5C—H5C	0.9300
C7A—C8A	1.518 (4)	C7C—C8C	1.528 (4)
C7A—H7A1	0.9700	C7C—H7C1	0.9700
C7A—H7A2	0.9700	C7C—H7C2	0.9700
C8A—C9A	1.521 (4)	C8C—C9C	1.517 (4)
C8A—H8A	0.9800	C8C—H8C	0.9800
C9A—C11A	1.570 (5)	C9C—C11C	1.558 (5)
C9A—H9A	0.9800	C9C—H9C	0.9800
C10A—C14A	1.498 (9)	C10C—C14C	1.520 (7)
C10A—C13A	1.520 (8)	C10C—C13C	1.525 (8)
C11A—C12A	1.510 (5)	C11C—C12C	1.494 (5)
C11A—H11A	0.9800	C11C—H11C	0.9800
C12A—H12A	0.9700	C12C—H12E	0.9700
C12A—H12B	0.9700	C12C—H12F	0.9700
C13A—H13A	0.9600	C13C—H13G	0.9600
C13A—H13B	0.9600	C13C—H13H	0.9600
C13A—H13C	0.9600	C13C—H13I	0.9600
C14A—H14A	0.9600	C14C—H14G	0.9600
C14A—H14B	0.9600	C14C—H14H	0.9600
C14A—H14C	0.9600	C14C—H14I	0.9600
S1B—O2B	1.403 (4)	S1D—O2D	1.410 (4)
S1B—O1B	1.430 (3)	S1D—O1D	1.422 (3)
S1B—C7B	1.766 (3)	S1D—C6D	1.757 (4)
S1B—C6B	1.766 (4)	S1D—C7D	1.760 (3)
O3B—C10B	1.428 (4)	O3D—C10D	1.391 (5)
O3B—C11B	1.429 (4)	O3D—C11D	1.418 (4)
O4B—C10B	1.427 (4)	O4D—C9D	1.412 (4)
O4B—C9B	1.428 (4)	O4D—C10D	1.440 (5)
O5B—N1B	1.432 (3)	O5D—N1D	1.439 (3)
O5B—H5BO	0.8200	O5D—H5DO	0.8200
N1B—C12B	1.473 (4)	N1D—C8D	1.471 (4)
N1B—C8B	1.481 (4)	N1D—C12D	1.471 (4)
C1B—C6B	1.372 (6)	C1D—C2D	1.380 (8)
C1B—C2B	1.384 (7)	C1D—C6D	1.385 (7)
C1B—H1B	0.9300	C1D—H1D	0.9300
C2B—C3B	1.347 (10)	C2D—C3D	1.372 (13)
C2B—H2B	0.9300	C2D—H2D	0.9300
C3B—C4B	1.329 (10)	C3D—C4D	1.339 (13)
C3B—H3B	0.9300	C3D—H3D	0.9300
C4B—C5B	1.406 (9)	C4D—C5D	1.386 (10)
C4B—H4B	0.9300	C4D—H4D	0.9300
C5B—C6B	1.356 (6)	C5D—C6D	1.356 (7)
C5B—H5B	0.9300	C5D—H5D	0.9300
C7B—C8B	1.540 (4)	C7D—C8D	1.529 (4)
C7B—H7B1	0.9700	C7D—H7D1	0.9700
C7B—H7B2	0.9700	C7D—H7D2	0.9700
C8B—C9B	1.502 (4)	C8D—C9D	1.522 (4)
C8B—H8B	0.9800	C8D—H8D	0.9800
C9B—C11B	1.563 (4)	C9D—C11D	1.560 (4)
C9B—H9B	0.9800	C9D—H9D	0.9800
C10B—C14B	1.505 (7)	C10D—C14D	1.496 (8)
C10B—C13B	1.511 (6)	C10D—C13D	1.531 (7)
C11B—C12B	1.499 (4)	C11D—C12D	1.508 (4)
C11B—H11B	0.9800	C11D—H11D	0.9800
C12B—H12C	0.9700	C12D—H12G	0.9700
C12B—H12D	0.9700	C12D—H12H	0.9700
C13B—H13D	0.9600	C13D—H13J	0.9600
C13B—H13E	0.9600	C13D—H13K	0.9600
C13B—H13F	0.9600	C13D—H13L	0.9600
C14B—H14D	0.9600	C14D—H14J	0.9600
C14B—H14E	0.9600	C14D—H14K	0.9600
C14B—H14F	0.9600	C14D—H14L	0.9600
			
O1A—S1A—O2A	115.6 (3)	O1C—S1C—O2C	116.9 (3)
O1A—S1A—C6A	107.1 (2)	O1C—S1C—C6C	108.1 (2)
O2A—S1A—C6A	109.0 (2)	O2C—S1C—C6C	108.9 (2)
O1A—S1A—C7A	108.0 (2)	O1C—S1C—C7C	108.0 (2)
O2A—S1A—C7A	108.44 (18)	O2C—S1C—C7C	108.11 (19)
C6A—S1A—C7A	108.54 (16)	C6C—S1C—C7C	106.37 (18)
C11A—O3A—C10A	109.0 (3)	C10C—O3C—C11C	108.7 (3)
C9A—O4A—C10A	108.3 (3)	C9C—O4C—C10C	107.4 (3)
N1A—O5A—H5AO	109.5	N1C—O5C—H5CO	109.5
O5A—N1A—C12A	109.9 (2)	O5C—N1C—C12C	110.2 (3)
O5A—N1A—C8A	110.3 (2)	O5C—N1C—C8C	111.2 (2)
C12A—N1A—C8A	105.8 (2)	C12C—N1C—C8C	106.1 (2)
C2A—C1A—C6A	119.3 (3)	C2C—C1C—C6C	120.1 (4)
C2A—C1A—H1A	120.4	C2C—C1C—H1C	120.0
C6A—C1A—H1A	120.4	C6C—C1C—H1C	120.0
C3A—C2A—C1A	120.2 (4)	C3C—C2C—C1C	119.9 (5)
C3A—C2A—H2A	119.9	C3C—C2C—H2C	120.0
C1A—C2A—H2A	119.9	C1C—C2C—H2C	120.0
C2A—C3A—C4A	120.5 (4)	C2C—C3C—C4C	120.8 (5)
C2A—C3A—H3A	119.8	C2C—C3C—H3C	119.6
C4A—C3A—H3A	119.8	C4C—C3C—H3C	119.6
C3A—C4A—C5A	120.4 (4)	C5C—C4C—C3C	119.3 (5)
C3A—C4A—H4A	119.8	C5C—C4C—H4C	120.4
C5A—C4A—H4A	119.8	C3C—C4C—H4C	120.4
C4A—C5A—C6A	119.3 (4)	C6C—C5C—C4C	120.5 (5)
C4A—C5A—H5A	120.4	C6C—C5C—H5C	119.8
C6A—C5A—H5A	120.4	C4C—C5C—H5C	119.8
C1A—C6A—C5A	120.2 (4)	C5C—C6C—C1C	118.5 (4)
C1A—C6A—S1A	124.4 (3)	C5C—C6C—S1C	115.5 (3)
C5A—C6A—S1A	115.3 (3)	C1C—C6C—S1C	125.5 (3)
C8A—C7A—S1A	115.0 (2)	C8C—C7C—S1C	114.8 (3)
C8A—C7A—H7A1	108.5	C8C—C7C—H7C1	108.6
S1A—C7A—H7A1	108.5	S1C—C7C—H7C1	108.6
C8A—C7A—H7A2	108.5	C8C—C7C—H7C2	108.6
S1A—C7A—H7A2	108.5	S1C—C7C—H7C2	108.6
H7A1—C7A—H7A2	107.5	H7C1—C7C—H7C2	107.6
N1A—C8A—C7A	112.3 (3)	N1C—C8C—C9C	99.9 (2)
N1A—C8A—C9A	100.5 (2)	N1C—C8C—C7C	112.3 (3)
C7A—C8A—C9A	112.5 (3)	C9C—C8C—C7C	112.8 (3)
N1A—C8A—H8A	110.4	N1C—C8C—H8C	110.5
C7A—C8A—H8A	110.4	C9C—C8C—H8C	110.5
C9A—C8A—H8A	110.4	C7C—C8C—H8C	110.5
O4A—C9A—C8A	111.1 (3)	O4C—C9C—C8C	110.3 (3)
O4A—C9A—C11A	103.7 (3)	O4C—C9C—C11C	104.2 (3)
C8A—C9A—C11A	104.7 (3)	C8C—C9C—C11C	105.0 (3)
O4A—C9A—H9A	112.3	O4C—C9C—H9C	112.3
C8A—C9A—H9A	112.3	C8C—C9C—H9C	112.3
C11A—C9A—H9A	112.3	C11C—C9C—H9C	112.3
O3A—C10A—O4A	103.8 (3)	O3C—C10C—O4C	104.3 (3)
O3A—C10A—C14A	109.5 (4)	O3C—C10C—C14C	111.3 (5)
O4A—C10A—C14A	109.0 (5)	O4C—C10C—C14C	110.2 (4)
O3A—C10A—C13A	109.4 (5)	O3C—C10C—C13C	107.5 (4)
O4A—C10A—C13A	110.1 (4)	O4C—C10C—C13C	108.5 (5)
C14A—C10A—C13A	114.4 (5)	C14C—C10C—C13C	114.6 (5)
O3A—C11A—C12A	110.5 (3)	O3C—C11C—C12C	109.8 (3)
O3A—C11A—C9A	104.1 (3)	O3C—C11C—C9C	103.3 (3)
C12A—C11A—C9A	105.0 (3)	C12C—C11C—C9C	105.3 (3)
O3A—C11A—H11A	112.2	O3C—C11C—H11C	112.6
C12A—C11A—H11A	112.2	C12C—C11C—H11C	112.6
C9A—C11A—H11A	112.2	C9C—C11C—H11C	112.6
N1A—C12A—C11A	102.0 (3)	N1C—C12C—C11C	102.1 (3)
N1A—C12A—H12A	111.4	N1C—C12C—H12E	111.4
C11A—C12A—H12A	111.4	C11C—C12C—H12E	111.4
N1A—C12A—H12B	111.4	N1C—C12C—H12F	111.4
C11A—C12A—H12B	111.4	C11C—C12C—H12F	111.4
H12A—C12A—H12B	109.2	H12E—C12C—H12F	109.2
C10A—C13A—H13A	109.5	C10C—C13C—H13G	109.5
C10A—C13A—H13B	109.5	C10C—C13C—H13H	109.5
H13A—C13A—H13B	109.5	H13G—C13C—H13H	109.5
C10A—C13A—H13C	109.5	C10C—C13C—H13I	109.5
H13A—C13A—H13C	109.5	H13G—C13C—H13I	109.5
H13B—C13A—H13C	109.5	H13H—C13C—H13I	109.5
C10A—C14A—H14A	109.5	C10C—C14C—H14G	109.5
C10A—C14A—H14B	109.5	C10C—C14C—H14H	109.5
H14A—C14A—H14B	109.5	H14G—C14C—H14H	109.5
C10A—C14A—H14C	109.5	C10C—C14C—H14I	109.5
H14A—C14A—H14C	109.5	H14G—C14C—H14I	109.5
H14B—C14A—H14C	109.5	H14H—C14C—H14I	109.5
O2B—S1B—O1B	119.8 (2)	O2D—S1D—O1D	118.3 (2)
O2B—S1B—C7B	108.65 (18)	O2D—S1D—C6D	108.3 (2)
O1B—S1B—C7B	106.76 (19)	O1D—S1D—C6D	108.4 (2)
O2B—S1B—C6B	107.9 (2)	O2D—S1D—C7D	108.06 (18)
O1B—S1B—C6B	107.96 (18)	O1D—S1D—C7D	107.1 (2)
C7B—S1B—C6B	104.84 (17)	C6D—S1D—C7D	106.10 (17)
C10B—O3B—C11B	108.4 (2)	C10D—O3D—C11D	108.2 (3)
C10B—O4B—C9B	108.5 (3)	C9D—O4D—C10D	108.1 (3)
N1B—O5B—H5BO	109.5	N1D—O5D—H5DO	109.5
O5B—N1B—C12B	110.0 (2)	O5D—N1D—C8D	110.7 (2)
O5B—N1B—C8B	111.2 (2)	O5D—N1D—C12D	109.8 (2)
C12B—N1B—C8B	104.9 (2)	C8D—N1D—C12D	105.4 (2)
C6B—C1B—C2B	120.9 (5)	C2D—C1D—C6D	119.7 (6)
C6B—C1B—H1B	119.5	C2D—C1D—H1D	120.2
C2B—C1B—H1B	119.5	C6D—C1D—H1D	120.2
C3B—C2B—C1B	118.5 (6)	C3D—C2D—C1D	118.3 (7)
C3B—C2B—H2B	120.8	C3D—C2D—H2D	120.9
C1B—C2B—H2B	120.8	C1D—C2D—H2D	120.9
C4B—C3B—C2B	121.6 (5)	C4D—C3D—C2D	121.7 (6)
C4B—C3B—H3B	119.2	C4D—C3D—H3D	119.1
C2B—C3B—H3B	119.2	C2D—C3D—H3D	119.1
C3B—C4B—C5B	120.8 (6)	C3D—C4D—C5D	120.8 (7)
C3B—C4B—H4B	119.6	C3D—C4D—H4D	119.6
C5B—C4B—H4B	119.6	C5D—C4D—H4D	119.6
C6B—C5B—C4B	118.3 (6)	C6D—C5D—C4D	118.4 (7)
C6B—C5B—H5B	120.8	C6D—C5D—H5D	120.8
C4B—C5B—H5B	120.8	C4D—C5D—H5D	120.8
C5B—C6B—C1B	119.7 (4)	C5D—C6D—C1D	121.2 (5)
C5B—C6B—S1B	121.6 (4)	C5D—C6D—S1D	119.1 (4)
C1B—C6B—S1B	118.7 (3)	C1D—C6D—S1D	119.7 (4)
C8B—C7B—S1B	114.1 (2)	C8D—C7D—S1D	114.8 (2)
C8B—C7B—H7B1	108.7	C8D—C7D—H7D1	108.6
S1B—C7B—H7B1	108.7	S1D—C7D—H7D1	108.6
C8B—C7B—H7B2	108.7	C8D—C7D—H7D2	108.6
S1B—C7B—H7B2	108.7	S1D—C7D—H7D2	108.6
H7B1—C7B—H7B2	107.6	H7D1—C7D—H7D2	107.6
N1B—C8B—C9B	101.0 (2)	N1D—C8D—C9D	101.3 (2)
N1B—C8B—C7B	111.8 (2)	N1D—C8D—C7D	112.4 (2)
C9B—C8B—C7B	114.6 (3)	C9D—C8D—C7D	113.4 (3)
N1B—C8B—H8B	109.7	N1D—C8D—H8D	109.8
C9B—C8B—H8B	109.7	C9D—C8D—H8D	109.8
C7B—C8B—H8B	109.7	C7D—C8D—H8D	109.8
O4B—C9B—C8B	109.8 (3)	O4D—C9D—C8D	111.0 (3)
O4B—C9B—C11B	103.7 (2)	O4D—C9D—C11D	103.3 (2)
C8B—C9B—C11B	105.5 (2)	C8D—C9D—C11D	105.1 (2)
O4B—C9B—H9B	112.4	O4D—C9D—H9D	112.3
C8B—C9B—H9B	112.4	C8D—C9D—H9D	112.3
C11B—C9B—H9B	112.4	C11D—C9D—H9D	112.3
O4B—C10B—O3B	103.3 (2)	O3D—C10D—O4D	104.0 (3)
O4B—C10B—C14B	109.3 (4)	O3D—C10D—C14D	108.8 (4)
O3B—C10B—C14B	108.2 (3)	O4D—C10D—C14D	109.6 (4)
O4B—C10B—C13B	110.0 (3)	O3D—C10D—C13D	110.6 (4)
O3B—C10B—C13B	110.7 (3)	O4D—C10D—C13D	108.5 (4)
C14B—C10B—C13B	114.6 (4)	C14D—C10D—C13D	114.7 (5)
O3B—C11B—C12B	110.5 (2)	O3D—C11D—C12D	110.5 (3)
O3B—C11B—C9B	103.7 (2)	O3D—C11D—C9D	104.2 (3)
C12B—C11B—C9B	104.6 (2)	C12D—C11D—C9D	104.7 (2)
O3B—C11B—H11B	112.5	O3D—C11D—H11D	112.3
C12B—C11B—H11B	112.5	C12D—C11D—H11D	112.3
C9B—C11B—H11B	112.5	C9D—C11D—H11D	112.3
N1B—C12B—C11B	101.6 (2)	N1D—C12D—C11D	101.9 (2)
N1B—C12B—H12C	111.4	N1D—C12D—H12G	111.4
C11B—C12B—H12C	111.4	C11D—C12D—H12G	111.4
N1B—C12B—H12D	111.4	N1D—C12D—H12H	111.4
C11B—C12B—H12D	111.4	C11D—C12D—H12H	111.4
H12C—C12B—H12D	109.3	H12G—C12D—H12H	109.3
C10B—C13B—H13D	109.5	C10D—C13D—H13J	109.5
C10B—C13B—H13E	109.5	C10D—C13D—H13K	109.5
H13D—C13B—H13E	109.5	H13J—C13D—H13K	109.5
C10B—C13B—H13F	109.5	C10D—C13D—H13L	109.5
H13D—C13B—H13F	109.5	H13J—C13D—H13L	109.5
H13E—C13B—H13F	109.5	H13K—C13D—H13L	109.5
C10B—C14B—H14D	109.5	C10D—C14D—H14J	109.5
C10B—C14B—H14E	109.5	C10D—C14D—H14K	109.5
H14D—C14B—H14E	109.5	H14J—C14D—H14K	109.5
C10B—C14B—H14F	109.5	C10D—C14D—H14L	109.5
H14D—C14B—H14F	109.5	H14J—C14D—H14L	109.5
H14E—C14B—H14F	109.5	H14K—C14D—H14L	109.5

### Hydrogen-bond geometry (Å, º)

**Table:**

*D*—H···*A*	*D*—H	H···*A*	*D*···*A*	*D*—H···*A*
O5*A*—H5*AO*···N1*B*^i^	0.82	2.04	2.752 (3)	144
O5*B*—H5*BO*···N1*A*^ii^	0.82	2.11	2.807 (3)	142
O5*C*—H5*CO*···N1*D*^i^	0.82	2.10	2.802 (4)	144
O5*D*—H5*DO*···N1*C*^ii^	0.82	2.06	2.766 (3)	144

Symmetry codes: (i) −*x*+1, *y*−1/2, −*z*+1; (ii) −*x*+1, *y*+1/2, −*z*+1.
